# Microbial Diversity Analysis of *Bupleurum latissimum* Nakai from Ulleung-Do of South Korea Using Next-Generation Sequencing

**DOI:** 10.1128/mra.01028-21

**Published:** 2022-03-16

**Authors:** Kyeung Min Han, Mi Na Choi, Jung Hun Pi, Sea Hyun Kim, Yeong Su Kim, Chae Sun Na

**Affiliations:** a Wild Plants and Seeds Conservation Department, Baekdudaegan National Arboretum, Chungyang-myeon, Bonghwa, South Korea; b Strategic Planning Division, Korea Arboretum and Gardens Institute, Sejong-si, South Korea; Montana State University

## Abstract

Bupleurum latissimum Nakai is a rare endemic species native to Ulleung-do in South Korea. This study aimed to report on the rhizosphere soil microbial diversity of B. latissimum. *Proteobacteria*, *Actinobacteria*, and *Verrucomicrobia* were identified in relative abundances of 27.77%, 21.70%, and 15.27%, respectively.

## ANNOUNCEMENT

Ulleung-do is an island with a total area of 73 km^2^, located 137 km east of the Korean Peninsula. It has a warm climate with several temperate plants; enlarged forms of Hepatica maxima, Valeriana dageletiana, and Bupleurum latissimum have been compared to the same species identified on the Korean Peninsula (1). In Ulleung-do, approximately 372 plant species are native and 40, including B. latissimum, are endemic (2). B. latissimum grows in the wild in a very steep area (inclination of 30° to 58°), 50 to 180 m above sea level on the northeast-northwest slopes of the valley near the sea, and prefers rocky soil with ample sunlight and ventilation (3). According to the evaluation of the International Union for Conservation of Nature (IUCN), B. latissimum is a rare plant classified as critically endangered (4, 5). To conserve such plants, a clear understanding of the basic biology of various species is important. Therefore, not only the environmental characteristics of Ulleung-do but also the soil microbial community need to be studied. This study aimed to analyze the diversity of the soil microbial community of B. latissimum using amplicon metagenomic sequencing.

Soil samples were collected on 17 June 2020 from Gyeongsangbuk-do, the native area of B. latissimum in Ulleung-gun in South Korea (Table 1). The upper part of the soil in which the plants were planted was removed by about 3 to 5 cm, and the soil was collected up to 20 cm from there, placed in an icebox, and stored at −20°C. DNA isolation used the DNeasy PowerSoil kit (Qiagen, Hilden, Germany). Next, 16S sequencing libraries were prepared using Herculase II Fusion DNA polymerase and the Nextera XT index kit v2, according to Illumina's instructions, with the target region (primer set) being V3V4 (Bakt 341F/805R primers) (6). PCR conditions were 3 min at 95°C for heat activation and 25 cycles of 30 s at 95°C, 30 s at 55°C, and 30 s at 72°C, followed by a 5-min final extension at 72°C. The prepared library was sequenced using the Illumina MiSeq platform of Macrogen, Inc. (South Korea). The 16S amplicon sequences were processed using DADA2 (7) and QIIME software v1.8.0, and diversity and taxonomic analyses were performed using amplicon sequence variant (ASV) sequences. Default parameters were used for all software unless otherwise noted. A summary of the coordinates of the sample collection sites and sequencing data is presented in Table 1.

**TABLE 1:** Collection sites and microbiome analysis read data

Collection point	Coordinates	No. of raw reads	No. of ASVs
Bl1	37.490083N, 130.903944E	200,500	1,237
Bl2	37.490028N, 130.903833E	187,200	1,220
Bl3	37.490086N, 130.903939E	196,200	1,324

**FIG 1:**
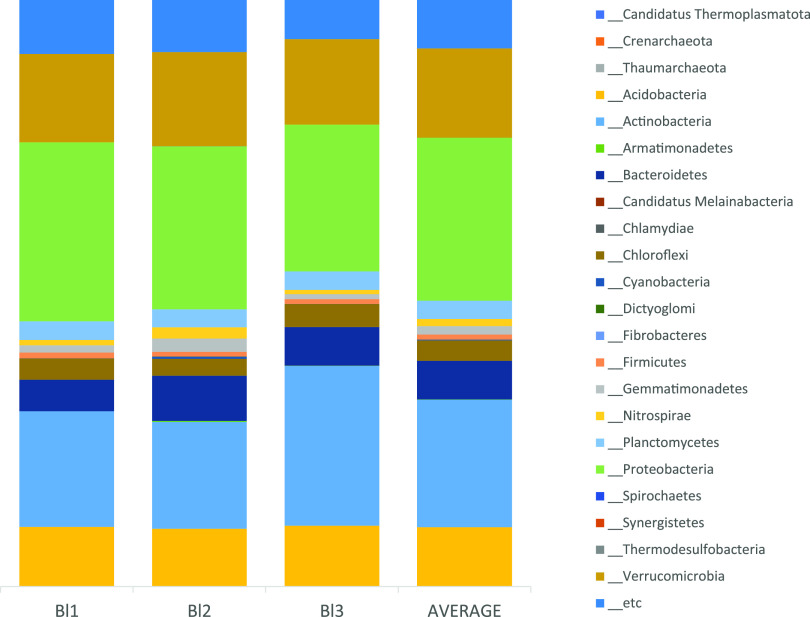
Relative abundances of the prokaryotic phyla in each position. Each color represents a different phylum.

Results revealed 280 prokaryotic families, 815 genera, and 1,845 species in approximately 22 phyla. *Proteobacteria* (relative abundance, 27.78%) were the most prevalent, followed by *Actinobacteria* (21.72%), *Verrucomicrobia* (15.24%), and *Acidobacteria* (10.12%), accounting for approximately 75% of the total population; the remaining phyla, including *Bacteroidetes*, accounted for 25% (Fig. 1).

This is the first report of the application of a sequencing method to a bacterial community with respect to a rare native plant, B. latissimum, and is expected to contribute to the development of cultivation and management methods in the future.

### Data availability.

The 16S rRNA gene amplicon sequences obtained from this study have been deposited in the Sequence Read Archive (SRA) of the NCBI database under the accession number SRX12798303.
